# Electronic Health Literacy Across the Lifespan: Measurement Invariance Study

**DOI:** 10.2196/10434

**Published:** 2018-07-09

**Authors:** Samantha R Paige, M David Miller, Janice L Krieger, Michael Stellefson, JeeWon Cheong

**Affiliations:** ^1^ Department of Health Education and Behavior University of Florida Gainesville, FL United States; ^2^ STEM Translational Communication Center University of Florida Gainesville, FL United States; ^3^ School of Human Development and Organizational Studies in Education University of Florida Gainesville, FL United States; ^4^ Department of Advertising University of Florida Gainesville, FL United States; ^5^ Department of Health Outcomes and Biomedical Informatics University of Florida Gainesville, FL United States; ^6^ Department of Health Education and Promotion East Carolina University Greenville, NC United States

**Keywords:** eHealth literacy, eHealth, aging, measurement invariance

## Abstract

**Background:**

Electronic health (eHealth) information is ingrained in the healthcare experience to engage patients across the lifespan. Both eHealth accessibility and optimization are influenced by lifespan development, as older adults experience greater challenges accessing and using eHealth tools as compared to their younger counterparts. The eHealth Literacy Scale (eHEALS) is the most popular measure used to assess patient confidence locating, understanding, evaluating, and acting upon online health information. Currently, however, the factor structure of the eHEALS across discrete age groups is not well understood, which limits its usefulness as a measure of eHealth literacy across the lifespan.

**Objective:**

The purpose of this study was to examine the structure of eHEALS scores and the degree of measurement invariance among US adults representing the following generations: Millennials (18-35-year-olds), Generation X (36-51-year-olds), Baby Boomers (52-70-year-olds), and the Silent Generation (71-84-year-olds).

**Methods:**

Millennials (N=281, mean 26.64 years, SD 5.14), Generation X (N=164, mean 42.97 years, SD 5.01), and Baby Boomers/Silent Generation (N=384, mean 62.80 years, SD 6.66) members completed the eHEALS. The 3-factor (root mean square error of approximation, RMSEA=.06, comparative fit index, CFI=.99, Tucker-Lewis index, TLI=.98) and 4-factor (RMSEA=.06, CFI=.99, TLI=.98) models showed the best global fit, as compared to the 1- and 2-factor models. However, the 4-factor model did not have statistically significant factor loadings on the 4th factor, which led to the acceptance of the 3-factor eHEALS model. The 3-factor model included eHealth Information Awareness, Search, and Engagement. Pattern invariance for this 3-factor structure was supported with acceptable model fit (RMSEA=.07, Δ*χ*^2^=*P*>.05, ΔCFI=0). Compared to Millennials and members of Generation X, those in the Baby Boomer and Silent Generations reported less confidence in their awareness of eHealth resources (*P*<.001), information seeking skills (*P*=.003), and ability to evaluate and act on health information found on the Internet (*P*<.001).

**Results:**

Young (18-48-year olds, N=411) and old (49-84-year olds, N=419) adults completed the survey. A 3-factor model had the best fit (RMSEA=.06, CFI=.99, TLI=.98), as compared to the 1-factor, 2-factor, and 4-factor models. These 3-factors included eHealth Information Awareness (2 items), Information Seeking (2 items), and Information and Evaluation (4 items). Pattern invariance was supported with the acceptable model fit (RMSEA=.06, Δ*χ*^2^=*P*>.05, ΔCFI=0). Compared with younger adults, older adults had less confidence in eHealth resource awareness (*P*<.001), information seeking skills (*P*<.01), and ability to evaluate and act upon online health information (*P*<.001).

**Conclusions:**

The eHEALS can be used to assess, monitor uniquely, and evaluate Internet users’ awareness of eHealth resources, information seeking skills, and engagement abilities. Configural and pattern invariance was observed across all generation groups in the 3-factor eHEALS model. To meet gold the standards for factor interpretation (ie, 3 items or indicators per factor), future research is needed to create and assess additional eHEALS items. Future research is also necessary to identify and test items for a fourth factor, one that captures the social nature of eHealth.

## Introduction

### Background

Telemedicine and electronic health (eHealth) transcends geographic, social, and political boundaries, making them essential tools to leverage health care delivery and surveillance [1,2]. The Internet has become deeply penetrated into society, with nearly 90% of adults in the United States having Internet access [3]. Millennials (18-35-year-olds), however, continue to adopt the Internet at a more rapid rate than members of Generation X (36-51-year-olds), Baby Boomers (52-70-year-olds), and the Silent Generation (71-115-year-olds) [4]. Although age-related disparities in Internet adoption have declined in recent years [5], the strategies to narrow this chasm and optimize the eHealth experience will require a closer look at the unique attributes of generations.

Generational differences in technological adoption can be broadly attributed to the point in one’s life that technology was penetrated into society [6]. Members of Generation X created the same technology that has become central to Millennials’ everyday lives. Rather than being familiar with and growing up with technology, Baby Boomers and members of the Silent Generation were introduced to technology after their social and cultural identities had been established. Widespread adoption of the Internet and the capabilities of technology have led Baby Boomers and members of the Silent Generation, who are traditionally considered late adopters of innovations like technology [4,6], to become excited and willing to adapt and learn about new technologies [7]. However, barriers related to unfamiliarity and uncertainty surrounding the use, value, and security of health information technologies persist among middle-to-older age adults [8-11], especially among those who are not avid health service users [12]. Evidence also shows that non-primary care physicians who are 55 years old and over are less likely to integrate electronic health record systems into their practice, as compared to their younger physician counterparts [13]. Consistent with theoretical underpinnings of the Diffusion of Innovation and the Technology Acceptance Model [7,14], technology tends to be adopted more quickly among younger age groups who find that it is both useful and easy to use.

Adoption of eHealth, however, does not ensure that the technology is used appropriately or that it is used to access high-quality and actionable health information [15]. eHealth literacy, driven by health and computer literacies, is defined as the capacity to locate, understand, evaluate, and act upon health information from technology [16]. People with a low degree of eHealth literacy are less likely to find the Internet as a useful health information tool, to trust the health information from diverse online sources and channels [17], and to actively seek out health information from the Internet [18]. Literacy in eHealth is a central skill set that influences not only health information seeking behaviors [19-21], but also the likelihood of engaging in proactive health-related outcomes and experiences [18,22]. Similar to generational values, researchers argue that social and cultural contextual frames influence eHealth literacy [23,24]. As such, understanding how generational age serves as a function of eHealth literacy and optimizing its measurement across these groups will be critical in the evolving technological era.

Empirical evidence over the past decade has shown that an inverse correlation exists between age and eHealth literacy [18,22,25,26]. Older adults generally have lower health literacy than their younger counterparts [27,28], yet this population is increasingly adopting the Internet with a high degree of confidence to access health information and supplement their health care [4,25]. Paige and colleagues [17] found that adults in the middle-to-older adult age groups, or Boomers and members of the Silent Generation, were more likely to have low eHealth literacy than their younger counterparts. Older age groups were also less likely to trust health information from social support forums but more likely to trust health information from Facebook. These age disparities have been attributed to older adults’ unique health needs as compared to younger adults [29,30], including specialized health information related to chronic disease [31-33], the potential risk for social isolation [34,35], and physical and cognitive limitations that are due to the natural aging process [36]. The generational differences in information seeking behaviors in the non-health context have also been highlighted in the literature to show that Millennials and Baby Boomers consult different informational sources [37]. For these reasons, it would be naïve to assume that eHealth literacy is measured and conceptualized equivalently across generational age groups. To our knowledge, evidence to support that measurement invariance of eHealth literacy scores exists across generations does not exist.

Valid age-group comparisons of eHealth literacy and associated patient-reported outcomes cannot be established without evidence that eHealth literacy measures function equally, or invariant, among young and old adults [38,39]. Measurement invariance indicates that the latent construct captured by an instrument will function similarly across different groups. Multi-group comparisons that do not meet the assumption of measurement invariance are ambiguous and subject to bias [38,40] and may use misleading or false claims to advance research and practice [41]. Without such evidence, it is unknown if the different relationship between eHealth literacy and age is due to real differences or systematic biases. As such, older adults may have a lesser degree of confidence to use eHealth. However, it is also possible that normal age-related cognitive declines [42] and low health literacy [28] readily reported among older adults contribute to depleted attention and working memory to recall accurate responses. As such, specific items may appear more salient in one age group over another. Establishing measurement invariance across eHealth literacy scales will have significant implications for fair and equitable testing standards. Also, it will alleviate bias in using these instruments to identify patients who are likely to benefit from online programs.

Since the conceptualization of eHealth literacy in 2006, several instruments have been developed to capture this construct in the evolving era of eHealth. The seminal instrument, the eHealth Literacy Scale (eHEALS), is a brief 8-item measure with theoretical underpinnings in self-efficacy, or the confidence in one’s capability to engage in behavior to result in the desired outcome [43]. Alongside the emergence of online social environments, like social media, there have been criticisms that the instrument has a compromised degree of content validity [16], particularly regarding its insufficient ability to capture the multidimensional and dynamic features of eHealth. In response, formative research was conducted to derive constructs salient to eHealth literacy and its measurement inductively. The most significant contribution noted by these instruments is the ability to capture the dynamic feature of eHealth and pressing issues related to eHealth use (eg, privacy). These instruments included items that assess if an Internet user can talk to their offline health care provider about the health information found on the Internet [44], as well as their skills related to privacy protection and message self-creation with a keyboard [45]. Another instrument related to eHealth literacy assessed the Internet users preferred mode of interaction and online experiences, as well as their degree of computer anxiety and health information needs [46]. These new instruments tap into unique aspects of eHealth literacy, but they do not provide insight into the communication exchange processes that are missing from eHEALS. Instead, these instruments have been said to leave eHealth literacy literature static, as recent attempts to advance the concept and measurement have not built upon previous literature [47]. Given this information, it is possible that new operational definitions, concepts, and measures that do not build upon seminal work of eHealth literacy may lead researchers astray from the core operational behaviors (ie, locate, understand, evaluate, act upon). Although measures of eHealth literacy have been published, eHEALS remains the most widely used and refined instrument in the literature [48-50] .

Evidence for the internal structure and external validity of eHEALS as a unidimensional measure exists across diverse age groups. These populations include adolescents [51], college students [52], the general adult population [52], patients with chronic disease [53], older adults recruited to surveys conducted online [54], and baby boomers and older adults recruited through the telephone [55]. More recently, studies applying sophisticated psychometric modeling techniques have found that eHEALS is a multi-dimensional measure that captures operational behaviors consistent with the seminal operational definition of eHealth literacy [16]. The eHEALS has been identified as a 2-factor measure of eHealth literacy among Australian adults who are at-risk for cardiovascular disease [56]. The 2-factor model was replicated among general adult populations in Germany [57] and Israel [57,58]. These factors have been defined as measuring information seeking and information appraisal. Most recently, the eHEALS 3-factor structure has been reported among adults later in the lifespan. Sudbury-Riley and colleagues [49] report that eHEALS scores produce a 3-factor model of information awareness, seeking, and appraisal skills among baby boomers. Similarly, the 3-factor model of eHEALS has been confirmed among baby boomers and older adults [55], chronic disease patients [53], as well as middle age adults, with an average age of 53, in a magnetic resonance imaging and computed tomography medical imaging outpatient clinic [59]. The 3-factor structure of eHEALS scores, however, has not been reported or confirmed among younger age groups, like Millennials or members of Generation X. As such, the discrepancy in 1-, 2-, and 3-factors captured among middle-to-older age adults and the general population in international contexts brings into question whether or not eHEALS produces similar factor structures across the lifespan.

Sudbury-Riley and colleagues [49] found measurement invariance for the 3-factor structure among baby boomers in the US, United Kingdom, and New Zealand. As such, the multidimensional eHEALS structure does not vary among Baby Boomers across international borders, regardless of the various health care provisions and coordination that drive social and cultural frames. Age, however, is also a strong determinant that shapes and influences the social and cultural frame of a given population [60]. Baby Boomers are a single generation within the lifespan, and measuring their health-related technological skills is well justified. However, Baby Boomers are a single generation whose socio-cultural and political frame has a significant influence on health outcomes and health services uptake [61-63]. Evidence that research on generational differences in eHealth adoption sets a precedent to also consider the potential generational variability in measures that assess eHealth literacy.

Although a “gold standard” eHealth literacy instrument does not exist [47], the eHEALS remains the closest to reaching this status due to its brevity, popularity, and theoretical underpinnings in health behavior change theory. Researchers have recommended the refinement of the eHEALS, specifically to account for the social nature of eHealth [47,64,65]. Before embarking on this mission, there is an obligation to understand the multidimensional factor structure of the eHEALS across age groups and whether or not these factor structures are invariant. Without such evidence, it will be challenging to refine eHEALS as a reliable measure that produces scores with a high degree of validity evidence across the lifespan in the social era of eHealth. Therefore, the purpose of this study is to examine the structure of eHEALS scores and the degree of measurement invariance among three generations: Millennials, Generation X, and Baby Boomers and the Silent Generation.

## Methods

### Sample and Procedures

A sample of Qualtrics Panelists from the United States completed an online survey in May 2015, which was approved by the university Institutional Review Board (IRB). The sample was stratified by race (ie, Caucasian, Black/African American). Per Qualtrics Panels, the survey functioned as opt-in, meaning that panelists who met inclusion criteria were offered the opportunity to consent to participate. For this particular survey, the inclusion criteria included residing within the United States and being older than 18 years old. Some respondents (n=11) did not provide their age and were subsequently removed from the final sample (N=829). Upon removing respondents who did not provide an age value, there were no missing eHEALS data in this sample.

### Measures

The following sociodemographic factors were measured across the sample [66]: (1) age (in years), (2) gender (Male, Female), (3) race (Black/African American, Caucasian), (4) ethnicity (Hispanic, non-Hispanic), (5) education level, (6) annual income, and (7) Internet use for Health. The eHealth Literacy was assessed with the eHEALS (Norman and Skinner, [51]), an 8-item, 5-point Likert-type rating scale (1=strongly disagree, 5=strongly agree).

### Data Analysis

The age group of the sample was categories as: (1) Millennials (18-35-year-olds), (2) Generation X (36-51-year-olds), and (3) Baby Boomers (52-70-year-olds) and Silent Generation (71-115-year-olds) [67]. Baby Boomers and the Silent Generation were collapsed into a single group for this study because the sample only contained 45 members of the Silent Generation. Frequency and descriptive statistics were computed to describe the sample and eHealth literacy scores. A series of chi-squared analyses were conducted to determine if sociodemographic factors were significantly different by age group.

#### Dimensionality

Multi-group exploratory structural equation models (MG-ESEM) were conducted with Mplus v7.3 [68] to inform the number of factors underlying eHEALS items. MG-ESEM is not a confirmatory factor analysis approach. Instead, it is a structural equation modeling (SEM) approach that integrates principles of exploratory factor analysis. This statistical approach is justified by the limited, and inconsistent (eg, 1-factor, 2-factor, 3-factor), knowledge regarding measurement properties of eHEALS across generations. For example, there is no priory theory to support that a certain number of factors are salient across generations who complete the eHEALS. Moreover, there is limited theoretical support to suggest that specific items from eHEALS would belong to one factor over another. Considered a novel framework to examine the measurement and structural properties through a SEM lens [69], a similar statistical approach has been used in measurement studies to examine the properties of eHEALS among baby boomers and older adults [55].

The fit of 4 MG-ESEM factor models was evaluated across each generation. Each model was independently examined, beginning with 1-, 2-, 3-, and finally a 4-factor model structure. The following criteria were used to evaluate the global model fit of each model [70]: (1) statistically non-significant chi-square value, (2) Root mean square error of approximation (RMSEA) value less than .08, (3) comparative fit index (CFI) value greater than .95, (4) Tucker-Lewis index (TLI) value greater than .95, (5) standardized root mean square residual (SRMR) less than .08, and (6) smaller Akaike information criterion (AIC). Factor loadings of .30 or greater [71] were examined to form the base model for testing measurement invariance.

#### Measurement Invariance

Mplus v7.3 [68] was used to carry out 3 analytical invariance tests within Confirmatory Factor Analyses (CFA) framework to test if the instrument functions similarly across each generational age group [39]. There were 3 tests of measurement invariance conducted [72]. The first was *configural invariance*, in which all parameters from the factor model identified in the MG-ESEM are freely estimated across groups to confirm that the underlying factor structure is equivalent*.* Next, *pattern invariance* tests the equivalence of unstandardized factor loadings across groups, which is used to examine if items are related to the factors in the similar ways across groups. Finally, the *unique invariance* test examines the equivalence of item measurement error across groups. Chi-square difference tests were conducted for model comparisons to test each level of measurement invariance, and several fit indices such as RMSEA, SRMR, CFI, and TLI, were also examined to evaluate the fit of the final model. A change in chi-square statistic was compared to the critical value with the relevant for the change in degrees of freedom*.* If the chi-square difference test was significant, adding invariance constraints was considered worsening the model fit and indicating lack of invariance. As chi-square is sensitive to sample sizes [73], a CFI change less than .01 was considered as non-significant changes in model fit, supporting invariance [74].

#### Comparing Electronic Health Literacy Scale Scores by Age Group

The statistical software SPSS v24 [75] was used to examine the internal consistency, or Cronbach’s alpha, of items comprising each eHEALS factor and compute the average of item scores. The reliability of each factor across age groups was determined by the omega coefficient, which is more appropriate for congeneric factor analysis models that do not function under tau-equivalence [76,77]. A one-way analysis of variance (ANOVA) and Tukey post-hoc analyses were conducted to identify the mean difference in eHealth literacy scores among each generation. Statistical significance was detected at *P*<.05.

## Results

### Sample Characteristics

As shown in Table 1, the mean ages of Millennials, Generation X, and Baby Boomers/Silent Generation Members were 26.64 (SD 5.14), 42.97 (SD 5.01), and 62.80 (SD 6.66), respectively. Respondents were mostly female (603/829, 72.74%), earning at least US $35,000 each year (499/829, 60.41%), and living with at least some college experience (623/829, 75.15%%). There were no statistically significant differences in gender, income, or education across each generation group. Nearly half of the respondents were Black/African American (412/829, 49.70%) or Caucasian (417/829, 50.30%), and most were non-Hispanic or Latino (807/829, 97.2%). A greater number of Millennials used the Internet for health-related purposes, as compared with members of Generation X or Baby Boomers/Silent Generation groups (*P*=.009).

### Dimensionality

The estimates of model fit for 1-4 factor models are presented in Table 2. Exceeding the acceptable level of RMSEA were the 1-factor (value=.14), 2-factor (value=.09) models. The 3-factor model (RMSEA=.06, 90% CI 0.04-0.08, CFI=.98, TLI=.98) and 4-factor model (RMSEA=.06, 90% CI 0.04-0.08, CFI=.99, TLI=.99) indicated good global model fit. Similarly, the AIC values for the 3-factor (value=12750) and 4-factor (value=12737.50) models were lower than the values for the 1-factor, 2-factor, and 4-factor models.

**Table 1:** Sociodemographics of millennials, generation X, and baby boomers/silent generation members.

Characteristic		Millennials (N=281)	Generation X (N=164)	Baby Boomers/Silent Generation (N=384)
Age in years, mean (SD)		26.64 (5.14)	42.97 (5.01)	62.80 (6.66)
**Gender, n (%)**				
	Male	73 (25.9)	54 (32.9)	99 (25.7)
	Female	207 (73.7)	110 (67.1)	286 (74.3)
	Missing	1 (0.36)	0 (0.0)	0 (0.0)
**Race^a^, n (%)**				
	Black/African American	156 (55.7)	93 (56.7)	163 (42.3)
	Caucasian	124 (44.3)	71 (43.3)	222 (57.7)
**Ethnicity, n (%)**				
	Hispanic	8 (2.9)	2 (1.2)	6 (1.6)
	Non-Hispanic	267 (95.4)	161 (98.2)	378 (98.2)
	Missing	5 (1.8)	1 (0.6)	1 (0.3)
**Education level, n (%)**				
	< High school	12 (4.3)	5 (3.0)	12 (3.1)
	High school/GED	58 (20.7)	31 (18.9)	88 (22.9)
	Some college	99 (35.4)	47 (28.7)	136 (35.3)
	Bachelor’s degree	65 (23.2)	39 (23.8)	67 (17.4)
	Master’s degree	26 (9.3)	28 (17.1)	59 (15.3)
	Advanced graduate	18 (6.4)	13 (7.9)	23 (5.9)
	Missing	2 (0.7)	1 (0.6)	0 (0.0)
**Annual income (US $), n (%)**				
	≤$20K/year	60 (21.6)	28 (17.1)	65 (16.9)
	$20K-$34,999K/year	62 (22.3)	33 (20.1)	79 (20.6)
	$35K-$49,999K/year	50 (18)	27 (16.5)	61 (15.9)
	$50K-$74,999K/year	57 (20.5)	25 (15.2)	88 (22.9)
	≥$75K more/year	49 (17.6)	51 (31.1)	91 (23.7)
**Internet use for health^b^, n (%)**				
	Yes	278 (99.3)	157 (95.7)	366 (95.1)
	No	2 (0.7)	7 (4.3)	19 (4.9)

^a^Black/African Americans and Caucasian respondents were less likely to be a member of Generation X than any other generation, *χ*^2^(2, N=829)=15.62, *P*<.001.

^b^More Millennials reported using the Internet for health, as compared to members of Generation X or Baby Boomers/Silent Generation, *χ*^2^ (2, N=829)=9.35, *P*=.009.

**Table 2:** Global model fit estimates for multi-group exploratory structural equation models

Model	*χ* ^2^ *(df)*	*P* value	RMSEA^a^ (90% CI)	SRMR^b^	CFI^c^	TLI^d^	AIC^e^
1-Factor Model	577.67 (90)	<.001	.14 (0.13-0.15)	.13	.88	.89	13135.05
2-Factor Model	263.16 (79)	<.001	.09 (0.08-0.10)	.09	.96	.95	12842.55
3-Factor Model	138.95 (67)	<.001	.06 (0.05-0.08)	.08	.98	.98	12742.33
4-Factor Model	108.12 (54)	<.001	.06 (0.04-0.08)	.08	.99	.98	12737.50

^a^RMSEA: root mean square error of approximation.

^b^SRMR: standardized root mean square residual.

^c^CFI: comparative fit index.

^d^TLI: Tucker-Lewis index.

^e^AIC: Akaike information criterion.

Although the 4-factor model yielded the best fitting model, items from the scale did not statistically significantly load onto the fourth factor. Therefore, the 3-factor model was used as the basis for assessing measurement invariance among young and old respondents.

**Figure 1:**
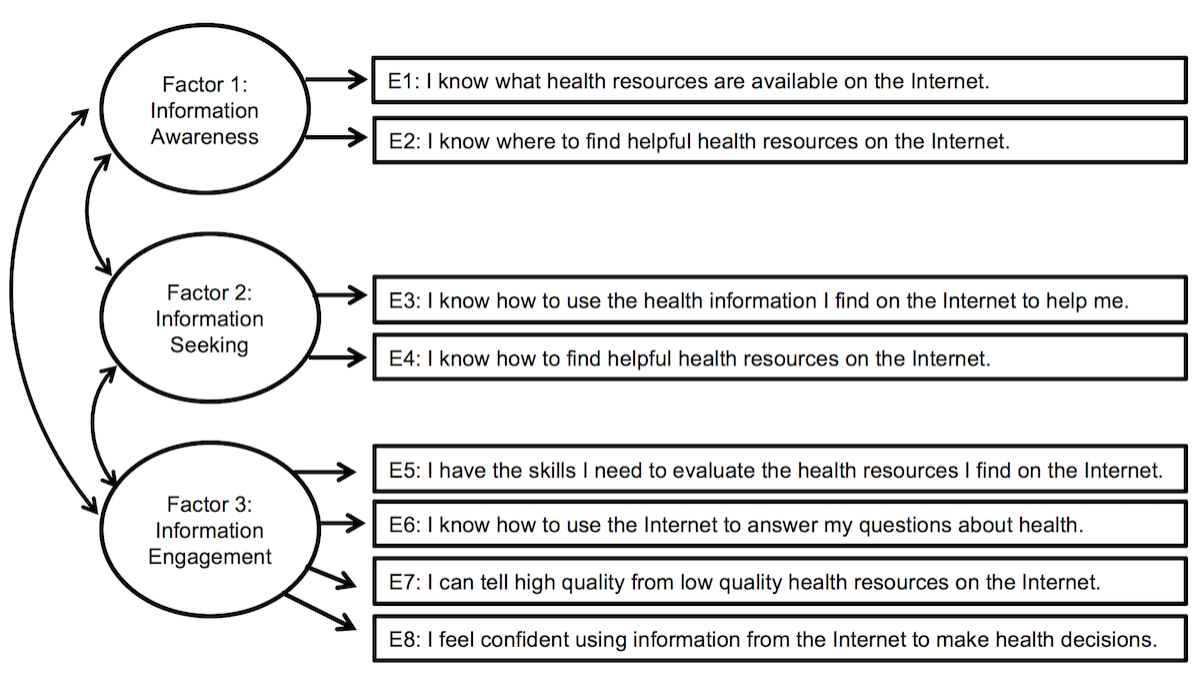
Proposed 3-factor electronic health literacy scale (eHEALS) measurement model.

Table 3 shows the statistically significant unstandardized factor loadings for the 3-factor model among Millennials, Generation X, and Baby Boomers/Silent Generation groups. Factor 1, which includes items that assess awareness about what health information is available on the Internet and where it can be located, contained significant factor loadings for Items 1-2 across all groups. Similarly, items 5-8 yielded high (greater than .40) and significant loadings on Factor 3, which included items that assess confidence in evaluating and using health information to answer health-related questions. Items 3 and 4, which assessed knowledge about how to use and find helpful health resource on the Internet, had a moderate to strong relationship with Factor 2 across all generation groups. All 3 factors were statistically significantly correlated with one another across all 3 groups. Interestingly, the correlation of Factor 1 with Factors 2 (*r*=.98) and 3 (*r*=.80) were much stronger than for the other generation groups. The final 3-factor model used to guide measurement invariance testing is shown in Figure 1.

**Table 3:** The 3-factor loadings for each generation.

Electronic Health Literacy Scale Item	Millennials^a^				Generation X^b^				Baby Boomer/Silent Generation^c^		
	Factor 1	Factor 2	Factor 3	Factor 1		Factor 2	Factor 3	Factor 1		Factor 2	Factor 3
(E1) I know what health resources are available on the Internet	.69^d^	.01	.06	.69^d^		.01	.06	.69^d^		.01	.06
(E2) I know where to find helpful health resources on the Internet	.64^d^	.18^e^	–.01	.64^d^		.18^e^	–.01	.64^d^		.18^e^	–.01
(E3) I know how to use the health information I find on the Internet to help me	.07	.39^f^	.22^f^	.07		.40^f^	.22^f^	.07		.40^f^	.22^f^
(E4) I know how to find helpful health resources on the Internet	–.02	.78^d^	.01	–.02		.78^d^	.01	–.02		.78^d^	.01
(E5) I have the skills I need to evaluate the health resources I find on the Internet.	–.09	.02	.72^d^	–.09		.02	.72^d^	–.09		.02	.72^d^
(E6) I know how to use the Internet to answer my questions about health.	–.01	.18^d^	.45^d^	–.01		.18^d^	.45^d^	–.01		.18^e^	.45^d^
(E7) I can tell high quality health resources from low quality health resources on the Internet	.17	–.03	.49^f^	.17		–.03	.49^d^	.17		–.03	.50^d^

^a^Factor 1 with Factor 2 (*r*=.70, *P*<.001), Factor 1 with Factor 3 (*r*=.63, *P*<.001), Factor 2 with Factor 3 (*r*=.76, *P*<.001).

^b^Factor 1 with Factor 2 (*r*=.98, *P*<.001), Factor 1 with Factor 3 (*r*=.80, *P*<.001), Factor 2 with Factor 3 (*r*=.77, *P*<.001).

^c^Factor 1 with Factor 2 (*r*=.79, *P*<.001), Factor 1 with Factor 3 (*r*=.79, *P*<.001), Factor 2 with Factor 3 (*r*=.89, *P*<.001).

^d^*P*<.001

^e^*P*<.05

^f^*P*<.01

### Measurement Invariance

Table 4 shows the results for configural, pattern, and unique factor invariance tests through the use of a CFA. The 3-factor model has slightly poorer, but acceptable, model fit in regards to configural invariance. This is determined from the RMSEA (value=.08, 90% CI 0.06-0.09) and CFI/TLI (.98 and .97, respectively), confirming that the 3-factor model represents the factor structure of eHEALS across all generations. Adding constraints on the factor loadings across generation groups (pattern invariance testing) resulted in slight improvement of RMSEA and relatively steady SRMR, CFI, and TLI values. The change in chi-square was not statistically significant and the CFI did not deviate by .01. In regard to unique factor invariance, the change in chi-square (Δχ^2^=69.51, Δ*df*=16) was statistically significant at. *P*<.05. As such, unique factor invariance was rejected as equating the error variances of each item across groups significantly diminished the model fit. Moreover, the AIC value for the pattern invariance model (value=12,770.60) was lower than the models testing for configural (value=12,775.72) and unique factor (value=12,808.11) invariance. Therefore, measurement invariance of for the proposed 3-factor structure exists among Millennials, the Generation X, and Baby Boomers/Silent Generation.

### Electronic Health Literacy Scale Scores by Age Group

Table 5 shows the average scale scores for the 3-factor eHEALS model across each generation. Internal consistency alpha estimates were within appropriate range for each factor, and omega coefficients demonstrated equivalent values to support reliability of the data. A one-way ANOVA showed that eHEALS scores varied across generations for Factor 1 (*F* [2, 827]=8.17, *P*<.001), Factor 2 (*F* [2, 826]=6.00, *P*=.003), and Factor 2 (*F* [2, 827]=18.51, *P*<.001). Tukey honest significant difference (HSD) post hoc analyses showed that, on average, members of the Baby Boomer and Silent Generation groups reported less knowledge and confidence in their eHealth literacy across all factors (*P*<.05), as compared to members of the Millennial and Generation X groups.

**Table 4:** Fit statistic summary for testing measurement invariance in the 3-factor model of electronic health literacy scale.

Model	χ^2^ (*df)*	RMSEA^a^	SRMR^b^	CFI^c^	TLI^d^	AIC^e^	Model comparison, Δχ^2^ ( Δ*df)*
Model 1: Configural Invariance	160.33 (61)	.08	.03	.98	.97	12775.72	0.0 (0)
Model 2: Pattern Invariance	175.21 (71)	.07	.05	.98	.97	12770.60	14.88 (10)
Model 3: Unique Factor Invariance	244.72 (87)	.08	.08	.96	.96	12808.11	69.51^f^ (16)

^a^RMSEA: root mean square error of approximation.

^b^SRMR: standardized root mean square residual.

^c^CFI: comparative fit index.

^d^TLI: Tucker-Lewis index.

^e^AIC: Akaike Information Criterion.

^f^*P*<.05.

**Table 5:** Average eHealth literacy scores by age group.

Electronic Health Literacy Scale Factor	Millennials			Generation X				Baby Boomer/Silent Generation			Total			
	α	ω	Mean (SD)		α	ω	Mean (SD)	α	ω	Mean (SD)^a^		α	ω	Mean (SD)
Factor 1: Information Awareness^b^	.80	.80	7.69 (1.68)		.91	.91	7.72 (1.80)	.83	.83	7.22 (1.67)		.84	.84	7.48 (1.71)
Factor 2: Information Seeking^c^	.86	.86	8.02 (1.50)		.90	.91	8.02 (1.57)	.89	.89	7.66 (1.46)		.88	.88	7.85 (1.51)
Factor 3: Information Engagement^d^	.79	.79	15.37 (2.66)		.85	.84	15.55 (2.77)	.86	.86	14.25 (2.97)		.84	.84	14.89 (2.88)

^a^*P*<.05.

^b^Factor 1 (min score=2; max score=10).

^c^Factor 2 (min score=2; max score=10).

^d^Factor 3 (min score=4; max score=20).

## Discussion

### Principal Findings

This study examined the degree of measurement invariance in eHEALS scores in the United States belonging to the Millennial, Generation X, and Baby Boomers/Silent Generations. The eHEALS is a multidimensional measure that can be used to assess eHealth literacy across the lifespan consistently. Millennials are more knowledgeable and confident in their online health information awareness, information seeking skills, and information engagement abilities, as compared to members of Generation X and the Baby Boomers/Silent Generation. Further, this study offers significant implications for the continued use and potential refinement of eHEALS in future research and practice-based settings.

The eHEALS scores best fit a positively correlated 3-factor model that captures the following underlying factors: *information awareness*, *information seeking*, and *information engagement*. This finding comes at a time when there is inconsistent evidence for the factor structure of eHEALS. Results of our study contrast with those described by Nguyen and colleagues [52], who explored the dimensionality of eHEALS when it was administered online to a significant proportion (60%) Millennials. Data from Nguyen and colleagues [52] showed eHEALS to have a unidimensional structure with a principal components analysis, which traditionally identifies the fewest number of factors that explain the substantial amount of variance in observed variables [78]. Considering the conflicting evidence describing the dimensionality of eHealth literacy, our alternative multi-group exploratory structural equation modeling approach sought to validate constructs implicit within eHEALS items across three different age groups. Moreover, the current study strived to cast a broader net to explore not only which eHEALS items best explained retained factors, but also how these factors might function in a theoretically driven manner consistent with eHealth literacy literature. Contrary to findings reported by Nguyen and colleagues [52], evidence generated in this study supported a 3-factor model of the English-version of eHEALS. Of note, our sample of Web-based panelists included proportionately more adults representing older Baby Boomer and Silent Generations. These 2 generations were underrepresented in their analyses conducted using an Internet-based sample obtained through machine learning software.

The 3-factor eHEALS model supported in this study captures a more precise assessment of eHealth literacy that goes beyond individual knowledge and perceptions of behavioral capability. The 3-factor eHEALS model comprises items that measure self-efficacy towards central operational skills related to eHealth literacy (ie, locate, evaluate, apply). These operational skills are associated with unique, albeit related, dimensions of self-efficacy in the context of eHealth literacy [16], which explains the highly correlated 3-factor model containing unique factors.

Configural and pattern invariance was upheld across all generation groups in the 3-factor eHEALS model, suggesting that eHEALS scores from the 3-factor model can be interpreted equivalently, regardless of respondents’ age group membership. Despite invariance between groups in the current study, the items that comprise each of these factors are inconsistent with the results of previous literature. Stellefson and colleagues [55] examined the factor structure of eHEALS scores among Baby Boomers and Silent Generation members during a telephone interview and found that Item 3 (ie, “I have the skills I need to evaluate the health resources I find on the Internet”) significantly loaded on both Factor 2 and Factor 3. In that study, Factor 2 included items related to knowing how to use and find helpful health information on the Internet to make informed health decisions. Factor 3 included only 1 other item with a significant factor loading, which addressed the ability to evaluate the quality of online health information. Findings from Stellefson and colleagues [55] run contrary to the current study and also findings reported by Sudbury-Riley and colleagues’ [49], who speculated that the content and theoretical underpinnings of this particular eHEALS item (Item 3) denote skills related to confidence in the ability to evaluate and act upon health information from the Internet. After a closer inspection of Item 3 content, it appears that this question may assess two distinct skills: (1) can one evaluate health information from the Internet? and (2) can one find health information on the Internet? The mode of data collection in Stellefson and colleagues’ [55] study was over the telephone, whereas the data collected in the current study and Sudbury-Riley and colleagues’ [49] was through a Web-based survey. It is possible that respondents only cognitively processed a single operational behavior outlined in this item (ie, find, evaluate), or perhaps the telephone interviewer placed emphasis on one skill over the other. Future research is needed to understand how data collection modality (eg, telephone, online) might directly affect the interpretation of the eHEALS items and ultimately the construct validity of the data produced.

Lastly, the final test of measurement invariance proved to be insufficient. The residual error variances of items were significantly different across age groups when tested within the 3-factor eHEALS model. Unique factor invariance is the strictest form of measurement equivalence. It is rarely achieved in practice, and experts have recently acknowledged that establishing unique factor invariance can be somewhat unreasonable for subjective measurement [79]. Therefore, we suggest that scores produced by the eHEALS may still be used as a comparative index to examine eHealth literacy across age groups [72].

### Limitations

This study sampled opt-in respondents from a Qualtrics Survey Panel taken from the general US population. Despite the population from which the sample was derived, the respondents were predominantly female with a normally distributed income and educational level. Moreover, half of the sample identified as Caucasian and the other half as Black/African American. In other words, this study enrolled over 400 respondents from population subgroups (ie, middle-older age adults, Black/African Americans) that are traditionally underserved in health promotion research. Although this represents a limitation affecting the generalizability of data to the entire US population, the diversity of sample characteristics remains a significant strength of this measurement study.

This was a self-reported Web-based survey, and, therefore, the results of this study can only speak to the interpretation and measurement invariance of scores from eHEALS administered on the Web. There is sufficient reliability and validity evidence of eHEALS delivered via telephone among middle-to-older age adults [55], a population most likely to respond differently to Web-based versus telephone-administered surveys [80]. Future research could explore the degree of measurement invariance of the 3-factor eHEALS model across generations according to the mode of survey administration. Moreover, respondents of this Web-based survey were members of Qualtrics Panels who opted-in to participate. The purposefully racially stratified sample and normally distributed income levels compromises the generalizability of the findings. However, the oversampling of minorities and low-income adults engaged these particularly vulnerable and hard-to-reach populations in survey research.

Although this study did not consider the geographic region (ie, rural versus urban) of the sample, nearly 70% of the sample reported using social media for health-related purposes, which requires a sufficient level of broadband. Rural adults are generally older [81] and have limited broadband connections [82] that enable sustained access to eHealth services. Moreover, rural residents are nearly twice as likely to not use the Internet as compared to their urban counterparts [82,83]. Therefore, factors beyond geographic location may limit rural adults’ eHealth use. Based on the limited empirical evidence related to the eHealth literacy of rural populations [84], future research is needed to explore eHealth literacy and its measurement among populations according to rurality regarding physical space (ie, Rural-Urban Commuting Area or Metropolitan Statistical Area data) and sociocultural rural identity.

### Practical Implications

Acknowledging the multidimensionality of scores obtained from eHEALS will allow practitioners to obtain a more precise understanding of consumers strengths and weaknesses using the Internet for health-related purposes. Rather than ambiguously interpreting “low eHealth literacy” based on prior unidimensional assumptions underlying eHEALS, practitioners considering the 3-factor model of eHEALS can identify the degree to which their patients have confidence in online health information awareness, search, and engagement. Interpreting scores based on 3 underlying eHEALS dimensions can assist practitioners and researchers to more efficiently direct patients to eHealth resources that are appropriate to their relative skill set, whether it is simply increasing awareness of existing online health information resources or providing a direct link to a particular website with credible health information. Precisely identifying limitations in core operational behaviors central to eHealth literacy will help to inform more tailored and efficient eHealth literacy interventions that consider an individual’s perceptions of technology adoption and acceptability.

Compared with Millennials, older generations reported lower knowledge and self-efficacy in each of the factors captured by eHEALS. Specifically, adults belonging to Generation X and the Baby Boomers/Silent Generation had less confidence in their (1) awareness of online health information, (2) skills to locate online health information, and (3) ability to evaluate and act on health information once it is located online. This finding is consistent with previous literature stating that older adults have lower proficiency in eHealth literacy than their younger counterparts [18,22,25,26]. However, it is currently difficult to measure the degree to which specific eHealth literacy skills are deficient across different age segments. Our study helps to shed light on how to interpret eHEALS scores, such that information is gathered regarding which particular eHealth literacy skills are limited and the degree to which they are limited across age groups. eHEALS has strong potential to be used as the standard assessment tool for coordinating eHealth literacy training interventions based on these three discrete factors. For example, structured interventions could be delivered in three modules where skill-building activities aim to improve eHealth awareness, as well as information seeking and evaluation. Although older and younger adults respond differently to eHealth literacy interventions [85], these 3 factors (ie, skill sets) are central components of eHealth literacy, and thus should be considered in the planning, implementation, and evaluation of training interventions designed to improve the eHealth literacy of older adults through narrowing the chasm that currently exists between eHealth adoption and sustained use.

Finally, the results of this study provide implications for refining and updating the eHEALS. The brevity of eHEALS makes it an ideal scale for use in research and clinical care. However, it is necessary to ensure that there is an adequate number of items that correspond to each factor. Some measurement guidelines support the reliability of highly correlated factors that only comprise 2 items each [86,87]. However, other measurement standards recommend including at least 3 items per factor [88]. In the current study, the 3 eHEALS factors were correlated to a statistically significant degree. The strong factorial relationship allowed the model to function adequately with fewer items on Factors 1 and 2. This finding is contrary to the findings reported by Sudbury-Riley and colleagues [49], who found that only 1 latent factor (ie, online health information awareness) was best reflected by 2 eHEALS items, whereas factors related to information seeking and application (eg, knowing how to find and use online health information, self-efficacy to evaluate, and use online health information) were comprised of 3 items each. Further research is needed to develop unbiased items that sufficiently capture the theoretical underpinnings of eHealth literacy and its multidimensional constructs. Moreover, to account for the dynamic and interactive nature of eHealth [2], future research can build upon our findings to create and test new items that account for a fourth latent factor that captures “social” skills related to eHealth literacy.

### Conclusion

Valid age group comparisons can be made with the 3-factor structure of eHEALS among Millennials, Generation Xers, and Baby Boomer/Silent Generation members. Results of this study add to the library of literature showing that older adults have significantly lower eHealth literacy scores as compared to younger adults. Specifically, this study supports that members of younger generations have a greater awareness of eHealth resources and more confidence in their information seeking and engagement skills on the Internet, as compared to older generations. The brevity of eHEALS coupled with its multi-dimensional structure can assist health care practitioners and researchers in tailoring eHealth literacy interventions designed to augment user performance on these relevant constructs. Furthermore, findings of this study have significant implications for more precisely measuring and improving eHealth literacy skills across the lifespan.

## Abbreviations

AIC: Akaike information criterion
CFA: confirmatory factor analysis
CFI: comparative fix index
eHEALS: electronic health literacy scale
IRB: Institutional Review Board
MG-ESEM: multi-group exploratory structural equation models
NIH: National Institutes of Health
NHLBI: National Heart, Lung, and Blood Institute
RMSEA: root mean square error of approximation
SRMR: standardized root mean square residual
TLI: Tucker-Lewis index
